# Physical, Chemical, and Enzymatic Pretreatment of Spent Hops and Its Impact on Xanthohumol Extraction Yield

**DOI:** 10.3390/molecules30102200

**Published:** 2025-05-18

**Authors:** Aleksandra Modzelewska, Mateusz Jackowski, Anna Trusek

**Affiliations:** Department of Micro, Nano and Bioprocess Engineering, Faculty of Chemistry, Wrocław University of Science and Technology, 50-370 Wrocław, Poland; mateusz.jackowski@pwr.edu.pl (M.J.); anna.trusek@pwr.edu.pl (A.T.)

**Keywords:** spent hops, xanthohumol, pretreatment methods, bioactive compounds, sustainable utilization

## Abstract

Spent hops from the supercritical extraction process represent a valuable source of xanthohumol (XN), a prenylated flavonoid with demonstrated anticancer, antidiabetic, antibacterial, and anti-inflammatory properties. However, XN is thermally sensitive and readily isomerizes into the less bioactive iso-XN at elevated temperatures, necessitating mild extraction conditions. Previous studies have shown that the pretreatment of plant biomass can enhance the extraction efficiency of bioactive compounds. In this study, various pretreatment methods—including physical (freeze–thaw, ultrasound, and microwave), chemical (acid and base hydrolysis), and enzymatic approaches—were applied to spent hops prior to extraction, and XN yields were compared to those obtained from untreated samples. The experiments, performed in triplicate, yielded meaningful results which helped understand the raw material’s behavior in applied conditions. Due to the compound’s high thermal sensitivity, ultrasound and microwave pretreatments require precise control to prevent excessive temperature increases, making low-temperature methods more suitable. Additionally, exposure to elevated pH adversely affected XN extraction efficiency, limiting the applicability of strong alkaline pretreatments. Among the evaluated methods, freeze–thaw pretreatment proved to be a simple and effective strategy, enhancing XN extraction yields by up to 10.7 ± 0.7% through the optimization of soaking time, the solid-to-liquid ratio, and the thawing temperature. Identifying an inexpensive and efficient pretreatment method could reduce extraction time while improving yield, contributing to the sustainable utilization of spent hops as an XN source.

## 1. Introduction

Hop cones (*humulus lupulus*) are widely applied in the brewing industry as one of the main components of beer, responsible for the beverage’s bitterness and aroma. Nowadays, numerous large breweries apply liquid hop extracts, which are produced by supercritical CO_2_ extraction [1]. Such extract is easy to dose and handle and affords better control over the production process, especially in automated brewhouses. A supercritical CO_2_ (scCO_2_) extraction process derives hop pellets from essential oils, α acids, and β acids. However, it leaves a variety of valuable compounds insoluble in scCO_2_, including xanthohumol [2].

According to Persistence Market Research, the global hop extract market is worth USD 1.632 mln. Among the products are scCO_2_ extracts, isomerized extracts, and oil extracts. It is estimated that scCO_2_ extracts are responsible for more than 50% of the market. The growth of the market is forecast at 3% annually. The main consumers of hop extracts are the food industry (especially the brewing industry) and the cosmetic industry [3,4]. Considering the growing production of hop extracts, it is desirable, from the point of view of the circular economy, to reuse byproducts from that branch of industry.

**Figure 1:**
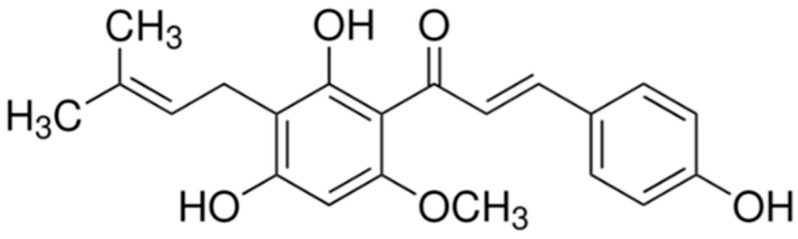
Chemical structure of XN.

Xanthohumol (XN) is a prenylated chalcone found only in hop plants (Figure 1) [5]. It has received much attention due to its biological effects and antidiabetic, antibacterial, or anti-inflammatory properties [5]. It also shows strong anticancer activity, including effects against leukemia, melanoma, lung, breast, cervical, colon, pancreatic, and gastric cancer [6,7]. Studies have also shown its anti-obesity activities and positive effect on glucose metabolism [8]. Spent hops are considered a valuable source of XN and numerous approaches were made to extract it most efficiently, including the use of solid/liquid extraction, pressurized-liquid extraction, microwave- and ultrasound-assisted extraction, or by applying emerging solvents, such as DESs (deep eutectic solvents) [9].

One of the biggest issues regarding the extraction and application of XN is its restricted stability. Temperature is a pivotal factor impacting XN’s stability, as higher temperatures accelerate its oxidation and degradation [10]; however, this phenomenon occurs even at temperatures as low as 4 °C. The degradation mechanisms include isomerization to a less active iso-xanthohumol (IXN), hydration, and ortho-position looping [11]. Thermal isomerization to IXN is the main reason behind the loss of XN during beer production, and only trace amounts of this compound are present in the final product [12].

Many studies performed on plant materials have proven a positive impact of sample pretreatment on the extraction yield. The most often applied methods include enzymatic, ultrasonic, pulsed electric field, infrared, or microwave pretreatment [13,14,15,16]. The overall aim of pretreatment processes for plant biomass is to modify the cellular structures while increasing the permeability of cell walls, therefore enhancing the availability and extractability of the demanded compounds [17].

**The ultrasonic treatment** of plant materials is an emerging green technology characterized by various advantages—it is cheap, does not consume a lot of time or energy, and does not generate pollution [17,18]. The application of ultrasonic waves (20–100 kHz) affects the extraction process both chemically and physically—collapsing microbubbles that are generated by the microwaves increases the mass transfer rate, the diffusion of the solvent into the matrix, and the surface area of the material, while the additional sonolysis of water molecules and the creation of free radicals cause cell disruption [19].

Ultrasound-assisted extraction (UAE) has found a large variety of applications in plant biomass processing. Data from the literature have shown numerous approaches to the extraction of oil, essential oil, polyphenols, protein, lipids, dyes, and pigments [18,20].

Factors affecting the efficiency of ultrasonic treatment are those connected to the equipment (frequency, power), solvent (viscosity, surface tension), and environmental parameters (temperature, pressure) [20]. High ultrasonic frequencies and temperatures accelerate the process but could destroy the structure of the demanded compounds. Lowering the ultrasound frequency, on the other hand, increases their stability while prolonging the process [21].

**Microwave treatment** is commonly used to obtain compounds from plant material—either as a pretreatment method or as the extraction step. The moisture retained within the biomass absorbs the photonic energy generated by electromagnetic waves, which causes the swelling and rupture of the plant cells and tissues. This results in an intensified release of plant compounds to the solvent. The application of microwaves requires only low energy consumption and is relatively fast [9]. Microwave treatment can be performed in two ways—in a closed vessel under high pressure (pressurized microwave treatment) or an open vessel under atmospheric pressure (focused microwave treatment) [22]. Microwave extraction is affected by the applied solvent system, microwave application time, microwave power level, temperature, and contact surface area [23].

In an experiment performed by Carbone et al. [24], microwave-assisted extraction using ethanol as a solvent resulted in an extraction yield reaching 19.1 mg of XN per 1 g of hop pellets, which was higher than any other method applied in this experiment. However, microwave energy absorption by the solvent leads to localized temperature rises, which must be considered when extracting thermolabile compounds, such as XN.

**Freeze–thaw** is a green and relatively simple method of solid sample treatment, where the cell walls and membranes are ruptured by the formation of ice crystals [25]. Freeze–thaw technique involves physically damaging cell walls by the growing ice crystals. For the process, the most important parameters are the rate of freezing and thawing, the temperature, and the pressure. It has been reported that this technique could damage cell membranes and cause lipid peroxidation and dehydration. In some cases, the gelation of biopolymers has also been observed [26,27]. This process requires soaking the sample in a proper solvent and cooling it to below the solvent’s freezing point. The freezing step is followed by thawing, usually performed at room temperature [28,29,30], but also in modified conditions—at lowered or elevated temperatures or with additional physical forces—high pressure, microwaves, or ultrasonic waves [31,32]. A properly adjusted freeze–thaw process makes it possible to obtain higher efficiency in the following processing steps of the materials, including the drying, extraction, pressing of oils, or their digestion [25,33,34,35].

Both the freezing and thawing temperature ranges influence the efficiency of further extraction. Numerous studies have shown a positive impact of applying large temperature differences between the freezing and thawing steps on the extraction of the demanded compounds from biomass [25,36]. It has also been proven that an extended soaking time before freezing can increase the extraction yield, as in the example of phycocyanin extraction from dry spirulina platensis powder [37]

Traditional freeze–thaw methods can be modified by incorporating other technologies, which aim to accelerate the process and, at the same time, reduce microbial reproduction during thawing [38]. In some cases, a shorter thawing time (obtained due to the application of additional techniques) leads to the improved integrity of food samples. Studies have shown that further extraction yields of various compounds from pre-treated products highly depends on modifications applied to traditional thawing processes [39].

**Microwave thawing** is a method in which microwaves are applied to frozen materials. This treatment overcomes the problem of heating the environmental medium before raising the temperature of the sample itself, while decreasing the thawing time [40]. However, it is crucial to control the process parameters carefully, as uncontrolled temperature rises may occur and degrade heat-sensitive compounds.

**Freeze-ultrasonic thawing technology** (FUTE) was proposed by [21] as an effective method for extracting anthocyanins from blueberry (*Vaccinium* spp.). FUTE makes it possible to extract significantly more anthocyanins (up to 2.53 mg/g) than ultrasound-assisted extraction (UAE) and freeze–thawing extraction (FTE) performed alone (1.25 mg/g and 1.01 mg/g, respectively). In this method, the extraction yield is strictly dependent on the liquid/solid ratio, freezing time, ultrasonic thawing time and temperature, and the number of freeze–thawing cycles.

**Enzymatic pretreatment**—the application of plant tissue-degrading enzymes is a method that increases the extraction yields of components from within the deeper plant structures and inside of the cells. The most commonly applied enzymes are cellulases, hemicellulases, proteases, and pectinases, which make it possible to enhance the extraction yields of pomegranate seed oil [41], *Rosmarinus officinalis* essential oil [42], proteins from quinoa [43], plum (*Prunus salicina* L.) juice [44], and many others. The most crucial parameters which determine the efficiency of the process are the enzyme/biomass ratio, temperature, time, and pH [45].

This research analyses the impact of the pretreatment methods mentioned above on the extraction yield of XN from spent hops, which were generated during supercritical CO_2_ extraction of hop extracts.

## 2. Results

### 2.1. The Influence of Time and Temperature on XN Extraction Yield

**Figure 2:**
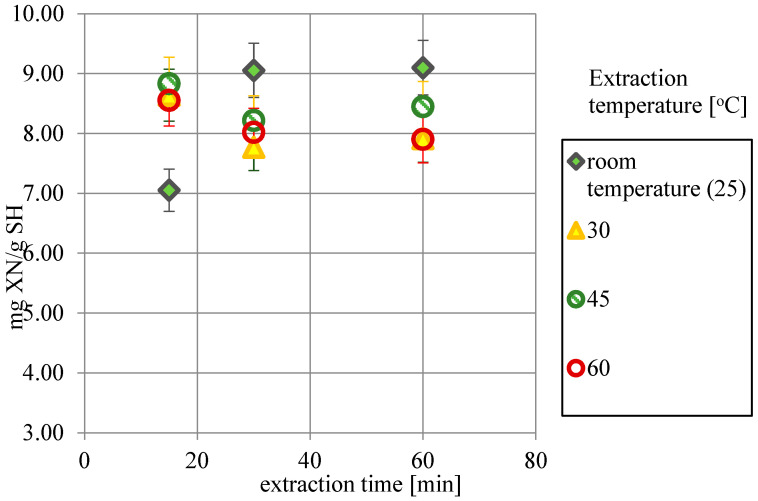
XN extraction yields obtained for different temperatures (25–60 °C) and time (15–60 min) for 50% isopropanol.

XN extraction yield from the untreated sample using 50% isopropanol (Figure 2) is greatly dependent on temperature. Elevating the temperature to 45–60 °C makes it possible to obtain relatively high extraction yields in 15 min; however, the concentration quickly decreases due to XN’s lack of thermostability and isomerization to iso-XN [11]. Room temperature gave the best results after 30 min of extraction, and the concentration in the sample remained unchanged for another 30 min. Due to the low energy consumption, extraction yield for 30 min at room temperature was chosen as the control value (9.05 mg of XN per 1 g of spent hops dry mass (mg_XN_/g_SH_)), and these conditions were also applied in extraction using the pretreated samples. 

### 2.2. Physical Pretreatment

#### 2.2.1. Ultrasonic Treatment

**Figure 3:**
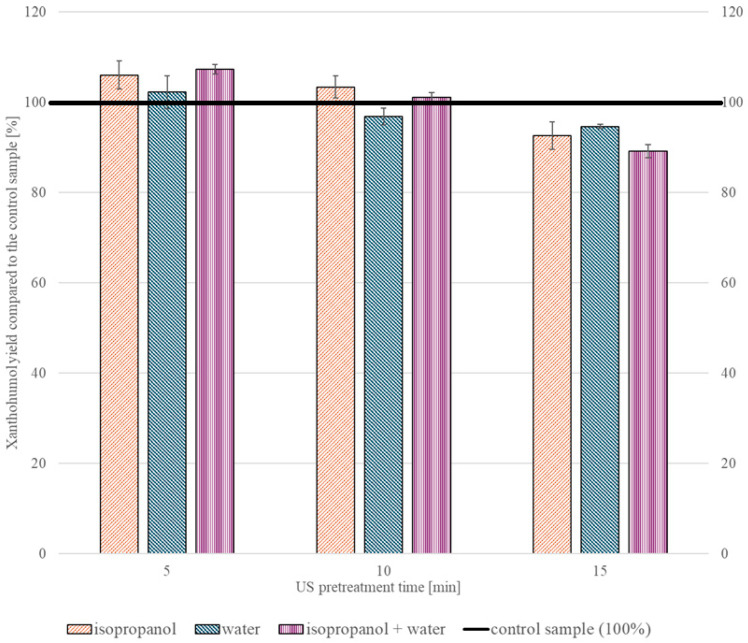
XN extraction yields obtained after ultrasonic pretreatment, using isopropanol and/or water as a solvent and treating the samples for 5–15 min.

The ultrasonic pretreatment of the samples (Figure 3) led to a slight increase in extraction yield when compared to the control samples. However, exceeding 10 min of pretreatment time led to a decrease in the extraction yield, reaching values below those of the untreated samples. Applying isopropanol and water as a solvent and 5 min of ultrasound gave the most promising results, exceeding the control sample by 7.3 ± 1.0%. 

#### 2.2.2. Microwave Treatment

Microwave pretreatment resulted in similar or lower amounts of XN compared to the untreated sample (Table 1). Using isopropanol as a solvent during microwave pretreatment slightly improved the extraction yield. However, the sample’s final temperature after 2 min of treatment was approximately 78 °C, which was close to isopropanol’s boiling point (82.3 °C); therefore, open-vessel pretreatment for a longer time or with higher microwave power was not possible. The same situation occurred in an isopropanol–water mixture, as the mixture’s boiling point was reached during the experiment. In general, the amount of XN extracted from microwave-pretreated samples is lower when compared to an untreated sample, most likely due to XN isomerization in high temperatures [46,47] or its degradation caused by microwaves.

#### 2.2.3. Freeze/Thaw

**Figure 4:**
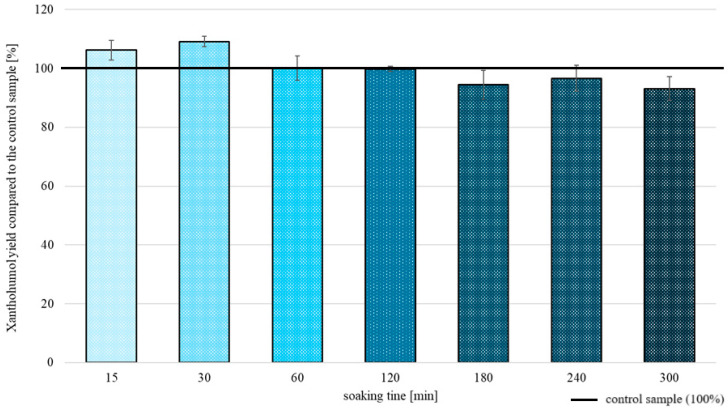
XN extraction yields obtained after freeze/thaw pretreatment obtained for different soaking times.

Extraction of XN from samples that underwent the freeze/thaw pretreatment was noticeably dependent on the soaking time (Figure 4). A period of 30 min of soaking led to the highest extraction yield (109.0 ± 1.7% in comparison to the control sample); whereas, lengthening the soaking time did not affect the process positively, with it showing a slow decrease in XN extraction yield.

**Figure 5:**
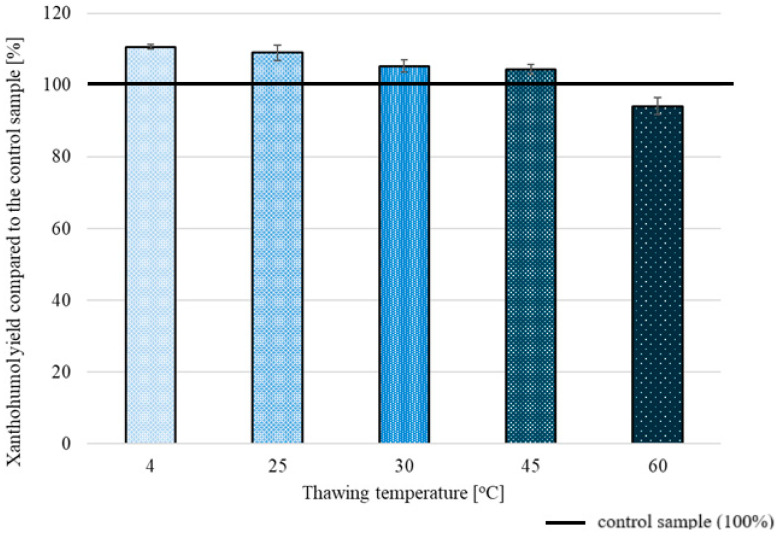
XN extraction yields obtained after freeze/thaw pretreatment for different thawing temperatures.

A more influential aspect affecting XN extraction yield was applying different thawing temperatures (Figure 5), which showed that thawing at 4 °C gave the most promising results, improving the extraction yield by 10.7 ± 0.7%.

#### 2.2.4. Ultrasound and Microwave Thawing

Ultrasound- and microwave-assisted thawing of the samples (Table 2) did not improve the XN extraction yield. The results were the highest for ultrasound-assisted thawing, which was almost the same as in the case of the untreated samples, and this was still lower than the result for the sample that was thawed at room temperature without additional force.

### 2.3. Chemical and Enzymatic Pretreatment

**Figure 6:**
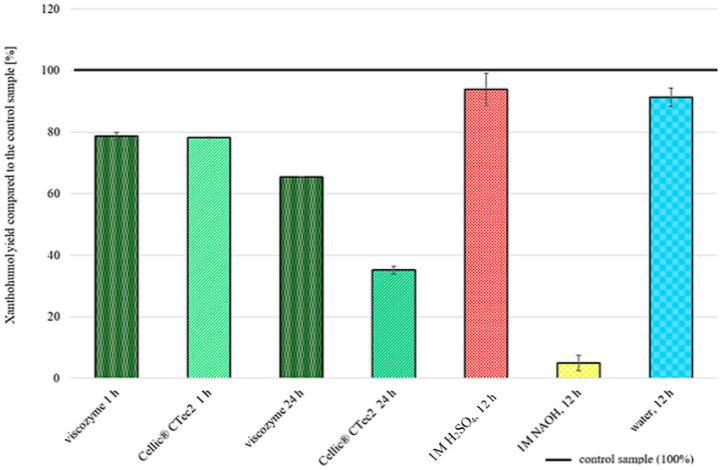
XN extraction yields obtained after chemical and enzymatic pretreatment.

**Figure 7:**
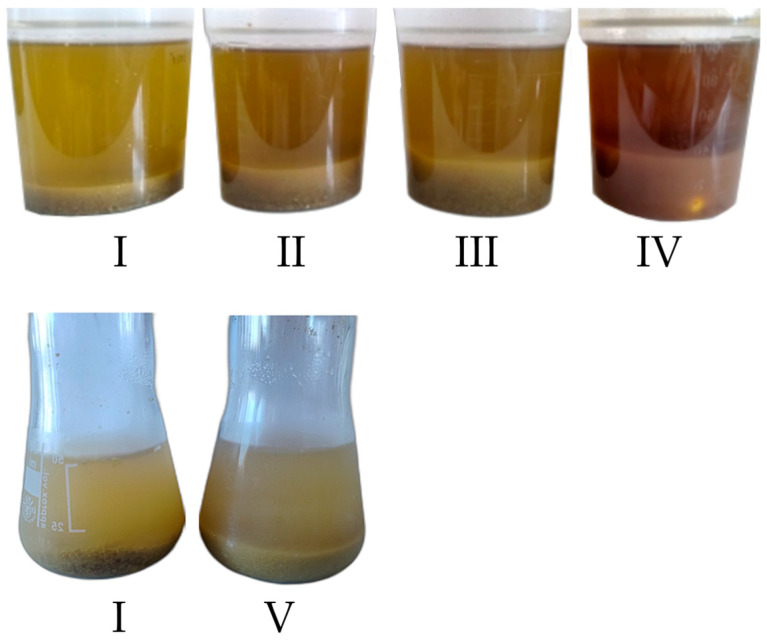
Visual comparison of samples treated chemically and enzymatically: I—control samples, II—water, III—H_2_SO_4_, IV—NaOH, V—Viscozyme L, each after 12 h of treatment.

The application of chemical or enzymatic pretreatment did not improve the XN extraction yield (Figure 6), even though the samples were visually different after pretreatment (Figure 7), showing a noticeable swelling of the biomass and, in the case of the NaOH-treated sample, a red color coming from the lignin reacting with the base [48]. The application of cell wall-degrading enzymes gave a lower yield than using no treatment at all. Soaking the sample in a H_2_SO_4_ solution gave similar results to using pure water (93.9 ± 5.2% and 91.3 ± 2.9%, respectively). Applying NaOH caused a drastic decrease in XN yield, which is caused by XN’s accelerated isomerization to iso-XN at an elevated pH [10].

## 3. Discussion

The physical pretreatment of spent hops from a supercritical extraction plant could improve XN extraction yield. The most promising results were obtained with the freeze/thaw method, where the thawing temperature was 4 °C. Thawing at this temperature requires a much longer time, along with the energy required for freezing the sample. The energy required for freezing biological materials may vary depending on the type of freezing device, the temperatures involved, the moisture content, etc. [49]. However, this method does not require specific installation or reagents, and storing frozen spent hops before extraction could slow down the degradation of XN in the sample caused by temperature and/or light exposure [11]. Studies on the impact of freezing and thawing temperatures on the plant material’s elastic and viscous toughness show that slow thawing at 4 °C lowers those factors [50]; therefore, cell wall degradation in those conditions promotes XN extraction. In the literature, slow thawing also enhanced the extraction of protein pertactin from *Bordetella pertussis* [51], as well as contributing to cell destruction in sunflower seeds [52] and microorganisms [53].

Due to the high temperature occurring in the microwave- and ultrasound-assisted treatment methods, which demanded a short time of application, low XN yields were extracted. The biggest obstacle is XN’s thermosensitivity, which causes its degradation in higher temperatures, including reactions like isomerization, hydration, and ortho-position cyclization [11]. The microwave-assisted treatment of the samples could be improved and give promising results, given that the samples are treated uniformly and cooling equipment is provided, as was shown in an experiment performed by Calinescu et al., 2017 [54], where polyphenols were extracted effectively from leaves of sea buckthorn by the additional application of a coaxial antenna and a cooling system.

No positive impact of enzymatic or chemical pretreatment could be caused by the fact that supercritical CO_2_ extraction modifies the cell wall matrix, which was proven by Femenia, A. et al. (2001) in a study considering almond fruits [55]. As a result, applying bases, acids, or enzymes might not contribute to the enhanced release of XN from already disrupted cells.

The physical pretreatment of spent hops before XN extraction could positively influence the extraction yield; however, it should be performed at low temperatures and without elevating the pH, due to XN’s quick degradation in high temperatures and basic environments. Processes like microwave or ultrasound treatment require additional cooling and mixing equipment to overcome this issue and improve extraction yields. While ultrasound-assisted processes are already being applied in the food industry [56,57], the freeze–thaw pretreatment of biomass prior to the extraction of specific compounds has not yet been fully introduced to large-scale application [58,59], and this study might contribute to its future utilization and expose the potential of this method. Although the freeze–thaw method requires a longer time than other pretreatment methods mentioned in this article, the equipment required is much less complex than ultrasound or microwave reactors. However, the thawing step could also be accelerated without the rise in the temperature brought about by the application of a proper low-temperature thawing system (high-pressure thawing, radio frequency thawing, etc.) [60].

## 4. Materials and Methods

**Figure 8:**
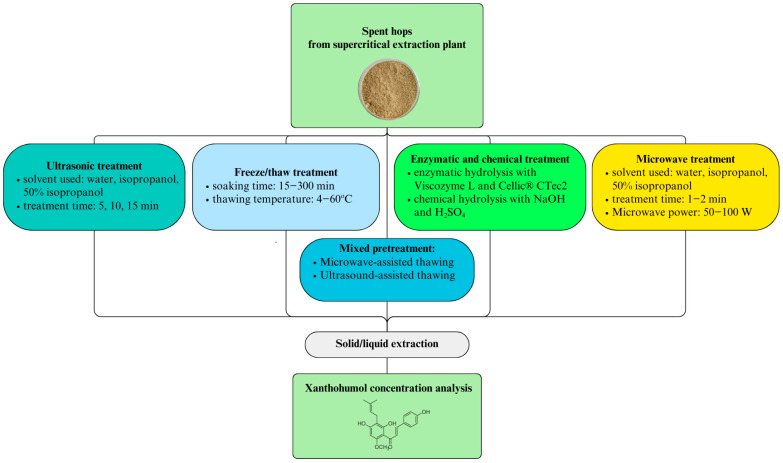
Experimental diagram.

In total, 1 g of spent hops “Magnum” (SH) from a supercritical carbon dioxide extraction plant (INS Łukasiewicz, Puławy, Poland), in the form of powder (92.2% dry mass) (Radwag MAC 110, Radwag Balances and Scales, Radom, Poland), was weighed with an accuracy of 0.05 g (Mettler Toledo AB104-S, Mettler Toledo, Columbus, OH, USA). Pretreatment methods (Figure 8) were later applied before XN extraction.

Physical pretreatment methods involved the addition of 50 mL of water and/or isopropanol to the sample. Microwave pretreatment was conducted by placing this in an open vessel in a microwave oven (constructed at Wrocław University of Science and Technology) for 1 or 2 min at 50 or 100 W. Ultrasonic pretreatment was performed in an ultrasonic bath (Bandelin, Sonorex, Berlin, Germany) operating at 35 kHz for 5–15 min.

The freeze/thaw method included soaking the samples for a given time (15–300 min) at room temperature and keeping them for approx. 12 h in the freezer at −19 °C. Afterward, the samples were thawed at different temperatures or by applying microwaves or ultrasound. Thawing temperatures were obtained in a water bath (25–60 °C) or a temperature-controlled refrigerator (4 °C). The process was performed until all ice crystals had disappeared, and the samples were mixed until they reached room temperature. In the case of microwave or ultrasound thawing, all samples were prepared by using 50 mL of water and soaking was performed for 30 min.

Chemical pretreatment was performed by adding 50 mL of 1 M NaOH (POCH) or H_2_SO_4_ (Chempur, Piekary Śląskie, Poland) solution to the sample, then stirring it for 12 h at room temperature. Enzymatic pretreatment was performed by placing the spent hops sample in 50% mL of acetate buffer solution and adding 1 mL of Viscozyme L or Cellic^®^ CTec3 (Sigma Aldrich, St. Louis, MO, USA). The pH was regulated according to the enzyme producer’s recommendations and was set at 4.3 and 5.05 for Viscozyme L and Cellic^®^ CTec3, respectively. The samples were stirred at 300 rpm for 12 h at room temperature. For comparison, one sample was prepared by applying pure water without acids, bases, or enzymes. Samples after chemical and enzymatic pretreatment were filtered before extraction.

XN extraction was performed for 30 min in a 1:1 mixture of water and isopropanol (50 mL + 50 mL), on a magnetic stirrer (300 rpm). This methodology was chosen based on previous experiments as it allows the extraction of high amounts of XN in mild conditions. The solvent mixture was obtained by adding appropriate amounts of water and/or isopropanol to previously treated samples. Control samples were obtained by the extraction of untreated spent hops in the same manner. Samples were centrifuged, and the supernatant was kept in a freezer until analysis. All experiments were performed in triplicate.

**Figure 9:**
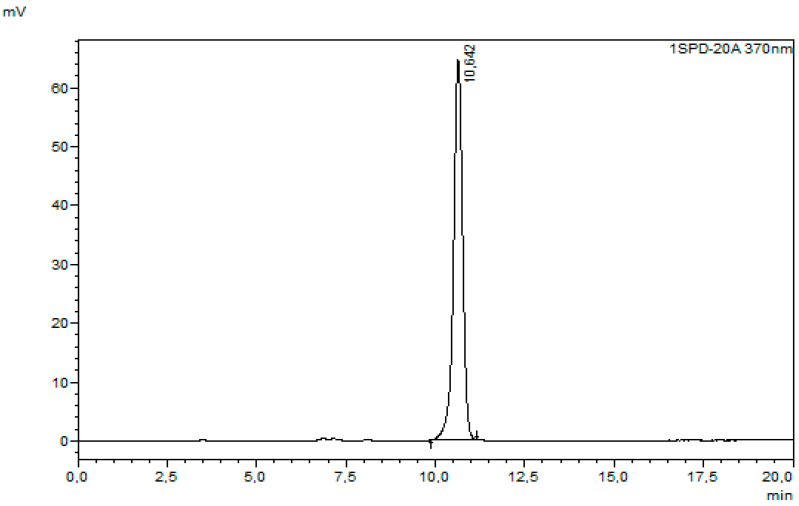
XN standard chromatogram.

**Figure 10:**
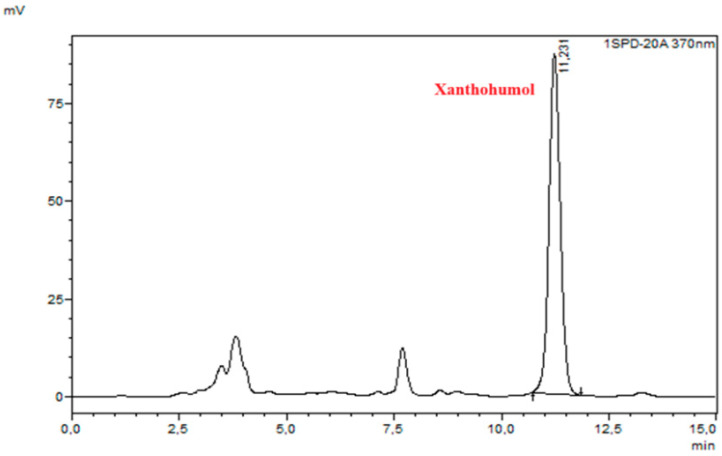
Control sample chromatogram.

The XN concentration in the obtained extracts was measured using HPLC with UV–vis detection (Shimadzu, Kyoto, Japan), with the mobile phase consisting of 85% methanol (HPLC grade, Chempur) and 15% water with formic acid (pH of aqueous phase = 2.6). The flow rate was set to 0.8 mL/min, and the injection volume to 10 µL. UV–vis detection was performed at 370 nm using a Fortis C18 LC column (5 µm, 250 × 4.6 mm). The XN from the hops (Sigma Aldrich) dissolved in methanol was applied to obtain a standard curve. The experimental results are expressed as mg of XN extracted per 1g of spent hop dry mass (mg_XN_/g_SH_) or as a percentage of the result for the control sample. Chromatograms of the XN standard sample and the control sample are presented in Figure 9 and Figure 10, respectively. 

## Figures and Tables

**Table 1:** Microwave pretreatment effects on XN extraction yields, using different solvents, microwave power, and treatment time.

**Microwave power [W]**	50		100	
**Treatment time [min]**	1	2	1	2
**Solvent composition:**	**XN extraction yield [% of control sample]**			
50 mL water	94.0 ± 0.3	100.1 ± 0.3	98.4 ± 0.5	95.8 ± 4.8
50 mL isopropanol	102.9 ± 1.0	Not performed due to solvent evaporation/mixture boiling		
50 mL water + 50 mL isopropanol	90.6 ± 5.9			

**Table 2:** XN extraction yields obtained after ultrasound and microwave thawing.

Thawing Method	Parameters	XN Extraction Yield
		[% of Control Sample]
**Ultrasound-assisted thawing**	15 min, 35 kHz	99.5 ± 2.2
	30 min, 35 kHz	99.2 ± 0.9
**Microwave-assisted thawing**	50 W, 8 min	86.8 ± 2.3
	80 W, 4 min	90.2 ± 6.7
	100 W, 3 min	89.9 ± 1.7
**Unassisted thawing at 25 °C**	109.02 ± 0.13	

## Data Availability

Data available within the article.
